# Diagnostic and Therapeutic Role of Peroneal Tenography in Chronic Peroneal Disorders: A Service Evaluation

**DOI:** 10.7759/cureus.81742

**Published:** 2025-04-05

**Authors:** Sandeep Munshi, Ranjith Nair, Rajiv Nair, Aditya Soni, Vaisakh Reghuram, Sivasankaran Munuswamy, Nadeem Baqai, Hariprasath Kanesan

**Affiliations:** 1 Trauma and Orthopaedics, Furness General Hospital, University Hospitals of Morecambe Bay NHS Foundation Trust, Barrow in Furness, GBR; 2 Trauma and Orthopaedics, University Hospitals of North Midlands NHS Trust, Stoke-on-Trent, GBR

**Keywords:** ankle mri, chronic ankle pain, lateral ankle imaging, peroneal tendon injury, tenography

## Abstract

Purpose

The aims were to investigate if there was a diagnostic difference between peroneal tenography and MRI to guide the management of peroneal tendon disorders with the secondary aim to investigate the therapeutic effect of peroneal tenography.

Methods

A retrospective study was carried out over a 75-month period, including all patients over 18 years who presented with ankle injuries to identify patients with peroneal tendon disorders. Symptomatic patients were investigated using MRI and peroneal tenography. This was also compared with intraoperative findings of peroneal tendons. Fischer's exact test was used to determine the diagnostic difference between peroneal tenography as compared to MRI. The therapeutic effect of peroneal tenography was also calculated.

Results

The cohort consisted of 27 patients (20 females and 7 males), with the median age being 50 years and the mean BMI being 31.9. The follow-up period after the final management plan was 12 months before discharge from care. Findings from peroneal tenography were more likely to match intraoperative findings than MRI when compared with intraoperative findings (p=0.033). Fifteen out of the 27 patients who underwent peroneal tenography reported adequate pain relief following the procedure.

Conclusion

Peroneal tenography is more sensitive and specific in determining peroneal tendon disorders as compared to MRI and plays a role as a therapeutic intervention in ankle pain management for these patients.

## Introduction

Approximately 2 million ankle sprains have been reported annually in the United States of America [1], and in the United Kingdom, approximately 14% of ankle sprains that were classed as severe in the accident and emergency unit resulted in ankle instability [2]. Hosack et al. found that most peroneal tendon injuries occur following trauma to the ankle [3]. These tendon injuries can go on to cause chronic peroneal tendon disorders, which commonly present as lateral ankle instability. They are believed to be a significant cause of posterolateral ankle pain [4]. The pathophysiology of peroneal tendon disorders includes inversion injuries, repetitive microtrauma from lateral instability, or tendinopathy associated with any systemic disorders such as diabetes mellitus, rheumatoid arthritis, collagen vascular diseases, and local steroid injections [5-6]. These can result in tendinopathy, tenosynovitis, partial and full thickness tendon tear, peroneal retinacular injuries, and tendon subluxation and dislocation.

Peroneal tendon instability occurs as a result of traumatic rupture of the superior peroneal retinaculum (SPR) following anterior force trauma along with peroneal contraction, with athletes being more prone to these injuries. Acute SPR tears are classified into three grades according to the Eckert and Davies classification [7]. A grade 1 tear is when the SPR is partially elevated off the fibula, a grade 2 tear is when the SPR is avulsed with the fibrocartilaginous ridge, and a grade 3 tear is when there is cortical avulsion with the SPR. A grade 4 tear was later described by Ogden in which the tear involves the avulsion of the SPR from the calcaneum, leaving the fibula intact [8]. Peroneus brevis is more prone to tear in chronic lateral ankle pain. Only 12.5% of patients with this chronic pain were found to have peroneus longus tendon tears, while 87.5% had a peroneus brevis tendon rupture [9].

A detailed history with a thorough clinical examination and adequate imaging studies are necessary for the diagnosis of peroneal tendon injuries and a high index of suspicion should be exercised for peroneal tendon tears in chronic ankle sprains [5]. Common imaging modalities for peroneal tendon injuries include magnetic resonance imaging (MRI), ultrasonography, and peroneal tendoscopy [4,10]. Compared to these imaging modalities, it is believed that peroneal tenography can provide more valuable information [11-12], as it has a sensitivity of 88% and a specificity of 87-94% for diagnosing tendon abnormality [13].

Tenography was initially found in 1970 by DG Palmer [14]. The use of peroneal tenography as a direct tool in the evaluation of peroneal tendon entrapment or displacement was described to be a safe and simple procedure with good predictable outcomes from as early as 1982 [15]. It was then improved in 1984 [16]. It is a minimally invasive technique of injecting radio-opaque dye into the peroneal tendon sheaths to examine the ankle under fluoroscopic guidance. It had been found to be useful as both a diagnostic and therapeutic procedure because the steroid is also infused into the tendon sheath as part of the procedure [11,13].

The introduction of ultrasound and MRI gained importance since the mid-1980s as non-invasive methods for detecting ankle and foot pathologies, and since then, tenography had been relegated to radiology history. However, Jaffee et al. observed its therapeutic value and published the first outcome analysis of tenography in 2001 [11].

At the time of this study, there were no studies that described the correlation between MRI and tenography findings in chronic peroneal disorders. The primary aim of this study was to compare the MRI and tenography findings in patients with chronic peroneal tear and tenosynovitis with the intraoperative findings, which is the gold standard to confirm the diagnosis [17]. The null hypothesis was that peroneal tenography and MRI have no diagnostic differences to guide the management of peroneal tendon disorders. The secondary aim was to study the therapeutic effectiveness of peroneal tenography for pain relief.

## Materials and methods

This retrospective study included all patients in this study center aged 18 years and over with peroneal tendon pathology over a 75-month period (January 1, 2015, to March 31, 2021). The study center is in the northwest of England, and it has a catchment population of approximately 365,000. The inclusion criteria were adults, 18 years and older, who underwent MRI of ankle and peroneal tenography. Patients who had previous foot and ankle trauma, surgeries and infection, and congenital and neuromuscular foot pathologies were all excluded from this study.

Patients with ankle injuries who matched the inclusion and exclusion criteria were retrospectively identified from the hospital electronic database. All patients' pain scores were assessed using the verbal rating scale [18], and it was measured as complete, partial, or no relief to allow the uniformity of responses throughout the study. All patients were initially trialled with conservative management, which included physiotherapy and a boot, for two weeks, and patients who failed to improve were reviewed in the fracture clinic with X-ray imaging. Patients with signs of acute peroneal injuries, which included tenderness over the retromalleolar area, positive active resisted eversion test, and Fleck sign on ankle X-rays [6], were investigated with foot and ankle MRI. The MRIs were reported by experienced musculoskeletal pathologists who classified the findings into three categories. The first category was tendinosis without tear, the second was tendinosis with peroneal brevis or longus or both tears, and the third was normal.

Patients with peroneal pathology and those with high suspicion of peroneal pathology visualised on MRI were offered peroneal strengthening exercises using TheraBands (Akron, OH, US). The peroneal tendon strength and ankle pain were assessed in follow-up visits, and patients were divided into three categories: good, mild, and no clinical improvement. Patients categorised into ‘good clinical improvement’ were encouraged to continue physiotherapy and were reviewed three months later in the clinic to assess progression. Patients with ‘mild’ and ‘no’ clinical improvement were offered peroneal tenography with steroid injection into the tendon sheath space. The results of the peroneal tenography were interpreted by two foot and ankle consultants who were highly experienced in this technique and classified the findings into five categories: normal, stenosis, degeneration, tear or rupture.

These patients were followed up in the clinic at six weeks and three months following tenography, and those found not to have improved symptoms were offered surgery for peroneal tendon exploration and repair. The intraoperative findings of patients who proceeded with the surgery were compared with their MRI and peroneal tenography findings to determine the accuracy of these investigations. The intraoperative findings were classified into three categories: peroneal tendon constriction, tenosynovitis or tendon tears.

Patient demographics were analysed for categorical variables. For the purpose of statistical analysis, the different sets of variables from the MRI, tenography and surgical findings were divided into normal and abnormal. Fischer's exact test was used to analyse the data comparing the MRI and tenography findings to the intraoperative findings. Pain relief post-MRI and tenography with intraoperative findings were analysed, and the differences were regarded as statistically significant when p < 0.05 [19].

## Results

This study group comprised 27 patients (females (N=20); Males (N=7)) who underwent peroneal tenography over a period of 6 years between January 2015 and March 2021. The median age of the cohort was 50 years, with a range from 29 to 80 years. Females underwent peroneal tenography approximately three times more often than males. The mean BMI of the cohort was 31.9. From the MRI report of the patients pre-procedure, normal MRI was reported in 48% of cases. Normal tenography findings were found in one-third of the cases, and 60% of cases were noted to have stenosis, which was indicated by constriction to the flow of the contrast material. Fourteen patients who did not have resolution of symptoms went on to have surgical management. In these 14 patients, the surgical diagnosis was found to be peroneal tear/split, mainly peroneus brevis (5 patients), tendon constriction (5 patients) and tenosynovitis (4 patients). None of these 14 patients were found to have normal tendons. These findings are displayed in Tables 1-3.

**Table 1:** MRI classification of patients included in the study Breakdown of MRI findings for included patients categorised into normal, peroneal tendon tear and no tear

MRI classification	Number of patients (n =27)
Tendinosis/tendinopathy without tear	7
Tendinosis/tendinopathy with tear	7
Normal MRI	13

**Table 2:** Tenography classification of patients included in the study Tenography findings categorised into normal, peroneal tendon stenosis and peroneal tendon split or degeneration

Tenography Classification	Number of Patients (n =27)
Stenosis	16
Longitudinal split/degeneration	2
Normal	9

**Table 3:** Intraoperative diagnosis of patients who had surgical management Intraoperative findings categorised into peroneal tear or split, constriction and tenosynovitis only.

Intraoperative Diagnosis	Number of Patients (n =14)
Tear/Split	5
Constriction	5
Tenosynovitis only	4

**Figure 1:**
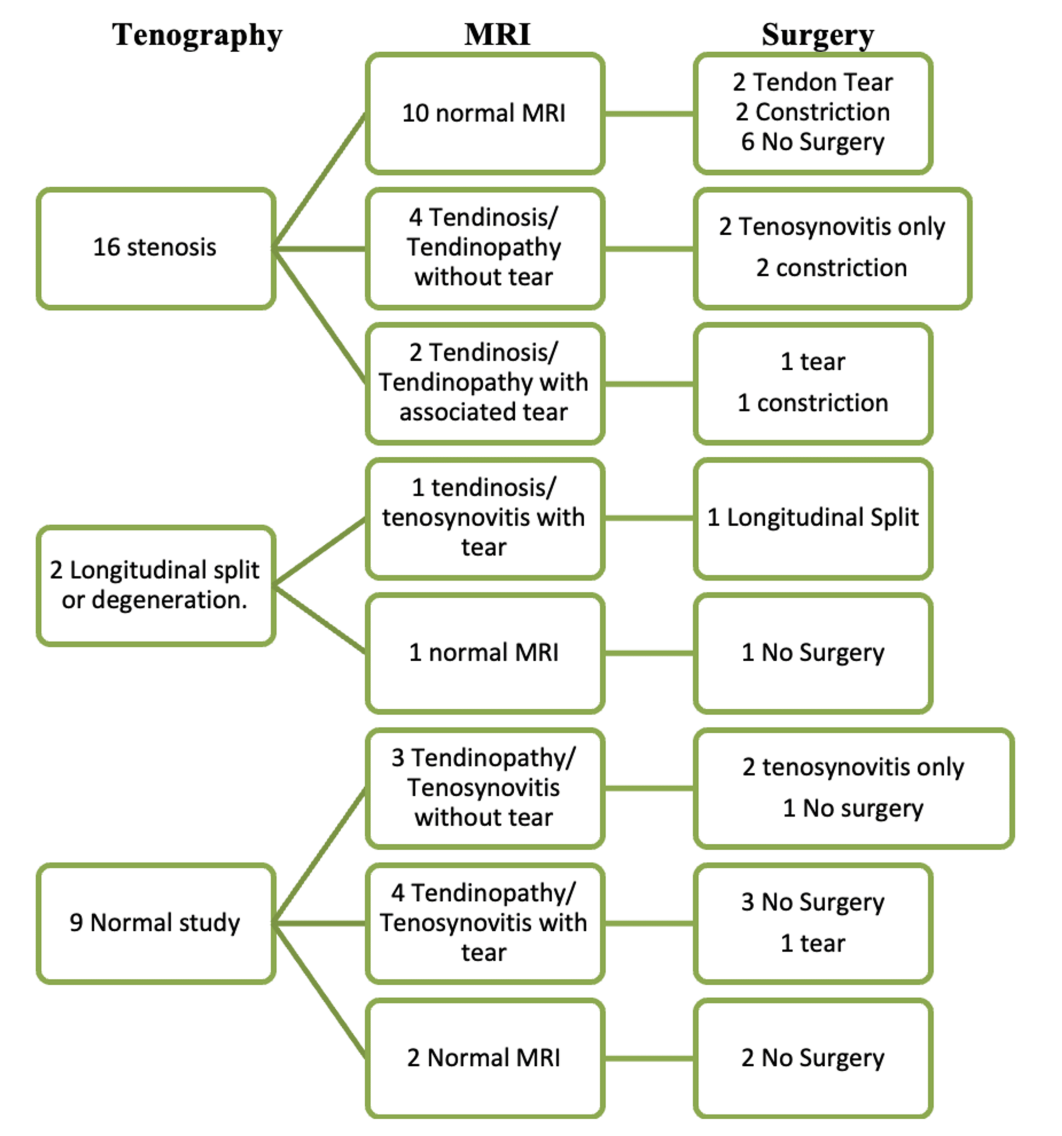
Relationship between MRI findings, tenography findings and intraoperative findings

A significant statistical correlation was noted between tenography and surgical diagnosis, indicating that its findings matched with the intraoperative findings (p=0.033), as seen in Figure 1. There could be a possibility of the data being skewed due to recall bias. This was because the surgeons performing the operation were aware of the peroneal tenography and MRI report prior to the procedure. Table 4 displays a comparison between the findings from peroneal tenography and intraoperative findings.

**Table 4:** Statistical comparison of tenography and intraoperative findings Tenography findings had a low rate of false positives and false negatives (Fischer's exact test p=0.033).

	Intraoperative Findings		
Tenography Findings	Abnormal	Normal	Total
Abnormal	11	0	11
Normal	1	2	3
Total	12	2	14

Importantly, the correlation of the MRI findings with the intraoperative findings among the cohort was not statistically significant. This indicated poor reliability of MRI compared to peroneal tenography to confirm the diagnosis (p=1). Table 5 demonstrates the statistical comparison between MRI and intraoperative findings.

**Table 5:** Statistical comparison of MRI and intraoperative findings All normal findings noted in MRIs from the cohort of participants were found to be false positives (Fischer's exact test p=1).

	Intraoperative Findings		
MRI Findings	Abnormal	Normal	Total
Abnormal	8	2	10
Normal	4	0	4
Total	12	2	14

Pain relief was found to be an added benefit of peroneal tenography. Fifty-six per cent (56%) of patients who underwent peroneal tenography reported partial to good pain relief. Table 6 shows the therapeutic effect of peroneal tenography for the cohort.

**Table 6:** Pain relief at eight weeks following peroneal tenography Fifty-six per cent (56%) of patients had partial to good pain relief 8 weeks following peroneal tenography.

Pain relief post tenography	Number of patients (n=27)	Percentage
No relief	12	44%
Partial relief	4	15%
Good relief	11	41%

When the tenography diagnosis was compared with the level of pain relief reported, no significant correlation was found (p=1). Table 7 compares the pain relief reported by patients to their individual tenography findings.

**Table 7:** Statistical comparison of pain relief and tenography findings Patients were more likely to have a partial to good extent of pain relief following peroneal tenography from both normal and abnormal tenography findings.

	Tenography Findings		
	Abnormal	Normal	Total
Pain relief			
Good to partial Relief	10	5	15
No relief	8	4	12
Total	18	9	27

Among patients who had an abnormal finding in their tenography, their pain following the procedure was as follows: eight patients reported no relief, three reported partial relief and seven reported good relief. In patients with normal tenography findings, four patients reported having no relief, one patient had partial relief and four patients reported good pain relief. From this, it is noted that peroneal tenography provided good to partial pain relief for approximately two-thirds of the cohort. Tables 8, 9 display the therapeutic effect of peroneal tenography in patients with normal and abnormal findings, respectively.

**Table 8:** Pain relief in patients with abnormal tenography Fifty-six per cent (56%) of patients reported some extent of pain relief following abnormal peroneal tenography findings.

Pain relief post abnormal tenography	Percentage (Number of patients), n=18
No relief	45% (8)
Partial relief	17% (3)
Good relief	38% (7)

**Table 9:** Pain relief in patients with normal tenography Fifty-five percent (55%) of patients reported some extent of pain relief following normal peroneal tenography findings.

Pain relief post normal tenography	Percentage (number of patients), n=9
No relief	45% (4)
Partial relief	10% (1)
Good relief	45% (4)

Patients with persisting lateral ankle pain after tenography were offered surgery to identify and treat the pathology. Patients with abnormal tenography findings were more likely to undergo peroneal surgery (61%) compared to patients with normal findings (11%). All of the patients who underwent surgery had good to excellent outcomes, and there were no post-operative complications reported up to the point of being discharged from follow-up, which was 12 months. Table 10 shows the percentage of patients who proceeded with surgery based on their pain relief post tenography. Tables 11, 12 compare the number of patients who proceeded to surgical management in those with normal and abnormal tenography findings, respectively, based on their pain relief. 

**Table 10:** Comparison between the pain relief post tenography and percentage of patients who proceeded with surgery Eighty-four per cent (84%) of patients with no pain relief, 50% with partial pain relief and 17% with good pain relief following peroneal tenography proceeded with surgical management of their pain.

Pain relief post tenography (Number of patients)	Proceeded with surgery (%)
No relief (12)	84%
Partial relief (4)	50%
Good relief (11)	17%

**Table 11:** Percentage of patients proceeding with surgery in those with abnormal tenography findings in accordance to their pain relief report One-hundred per cent (100%) of patients with no pain relief, 67% of patients with partial pain relief and 43% of patients with good pain relief following abnormal peroneal tenography findings proceeded to surgical management of their symptoms.

Pain relief in ABNORMAL tenography findings	Number of patients	Number of patients proceeding to surgery (percentage)
No relief	8	8 (100%)
Partial relief	3	2 (67%)
Good relief	7	3 (43%)

**Table 12:** Percentage of patients proceeding with surgery in those with normal tenography findings in accordance with their pain relief report Twenty-five per cent (25%) of patients with no pain relief and normal peroneal tenography findings proceeded to surgical management of their symptoms.

Pain relief in NORMAL tenography findings	Number of patients	Number of patients proceeding to surgery (percentage)
No relief	4	1 (25%)
Partial relief	1	0 (0%)
Good relief	4	0 (0%)

## Discussion

Lateral ankle pain has been found to be one of the most common presenting complaints in patients following inversion injuries to their ankles. In this patient cohort, there should be a high index of suspicion for peroneal tendon injury due to the mechanism of injury [20]. If left untreated, it could lead to chronic peroneal injuries, which would result in ankle instability, thus resulting in severe disability and disrupting the quality of life. Despite having imaging modalities, such as MRI and ultrasound scans, it has been proven to be challenging to accurately identify peroneal tendon injuries in these patients [21].

This study offered these patients peroneal tenography as an intermediary solution prior to suggesting surgical intervention. Although the study was unable to statistically demonstrate the therapeutic value of peroneal tenography, it found that there was a significant proportion of patients who benefited in terms of pain relief following tenography. Peroneal tenography did not increase the complexity of surgical management, but instead, it provided surgeons a better understanding of their patients’ condition prior to the operation.

Although peroneal tenography has significant benefits, as mentioned above, its risks should not be overlooked either. Although only a small percentage, these risks include infection, nerve block, bleeding, tendon injury, hypopigmentation and contrast allergy, and nephropathy [22]. There have been case reports published in which tendon degeneration and rupture were reported following injection of steroid into the tendon itself [23]. Steroids are believed to cause tendon degeneration by inhibiting tendon repair and delaying the healing of the tendon sheath due to the collagen fragmentation and biochemical changes caused [24]. 

In an attempt to prevent tendon damage, steroids were injected into the space within the tendon sheath after confirming the position of the needle by contrast and radiographs in this study. Dilution of contrast was observed following the steroid and local anaesthetic injection, which reconfirmed the position of the needle in the space. This enabled the minimisation of the risk of tendon rupture or further degeneration. A study by Jaffee et al., in 2001, reported only one case of tibialis posterior tendon rupture out of the 111 ankle tenography cases that were conducted [11]. In this study, there were no complications noted following peroneal tenography.

This study was unique, as it compared the effectiveness of MRI and peroneal tenography with the surgical diagnosis of patients, which is the gold standard to identify peroneal tendon pathology [25]. The study found that 48% of the cohort had normal MRI, but only 33% went on to have normal tenography. MRI demonstrated a similar incidence of tendinosis with tear and without tear (26%) to the tenography findings. However, stenosing tenosynovitis was found to be more accurately diagnosed by peroneal tenography (60%), and these patients were found to be more likely to undergo surgical management (10 out of 16 patients).

The different terms used in the MRI and tenography reports were noted and grouped to be better compared with intraoperative findings in an attempt to reduce the observational error. Although there was a possibility of intra- and inter-observer variability in tenography reporting and intra-operative findings, a clear demarcation was observed between the normal and abnormal findings in tenography when compared to MRI. This showed that tenography produced a better overview for surgical planning with the higher sensitivity of results.

The study observed good pain relief in 41% of the patients who underwent peroneal tenography compared to 46% of patients in the study done by Jaffee et al. in 2001 [11]. Although no statistical correlation was noted between tenography and pain relief, good to partial pain relief was reported in more than half of the patients who underwent tenography. It was also identified that patients with abnormal tenography findings required more surgical management of their symptoms than those with normal findings (62% vs 12.5%). This highlights that peroneal tenography is a sensitive investigation modality to help determine patients who would benefit from surgery. It has been reported previously that tenography provided a more consistent diagnosis as compared to MRI [26]. The sensitivity and specificity of peroneal tenography as a diagnostic and therapeutic modality make it a useful adjunct prior to surgical management, especially in cases where there is doubt in the diagnosis.

This study found a sensitivity of 92% for peroneal tenography in diagnosing peroneal pathology. Previous studies have found preoperative MRI to only have a sensitivity of 61.11% in diagnosing peroneal pathology [27]. This proves that surgeons should be more cautious when ruling out peroneal pathology in patients with chronic lateral ankle pain who have a normal MRI but have not had a tenography [27].

Other studies that have found MRI to have a sensitivity of 83.9% to identify peroneal pathology have also found that this percentage dropped significantly to 54.5% for identifying peroneal tears [12]. The sensitivity of MRI to identify peroneal tendon pathologies in this study was found to be 67%, which is in keeping with previous studies. There is an associated risk of radiation to the surgeons in performing peroneal tenography as compared to MRI or ultrasound. Ultrasound-guided peroneal sheath corticosteroid injection is a safe, non-invasive alternative to peroneal tenography for the diagnosis and treatment of peroneal tendon disorders. However, the diagnosis of peroneal tendon dislocation and tendinopathy using ultrasound has been found not to be very helpful in the diagnosis of stenosing tenosynovitis [28].

The diagnostic test to identify peroneal tendon pathology was conducted by instilling contrast material into the tendon sheath. This study was able to demonstrate the extrinsic compression or displacement of peroneal tendons following calcaneus fractures as a source of pain. It showed the ability to selectively anaesthetize the peroneal tendon sheath via proximal injection into the tendon sheath. Although this technique was considered to be a useful method to diagnose peroneal tendon disorders, it has been previously found that extravasation of contrast material and incomplete sheath filling could produce up to 10% false positive and 5% false negative results [28-29].

A prospective study carried out by Bleichrodt et al. in 1989 to assess the value of tenography in peroneal tendons for the diagnosis and classification of lateral ankle ligament ruptures found that tenography was reliable in the diagnosis of these tendon ruptures. Tenography was found to have a sensitivity of 88% and a specificity of 87-94% [13]. The positive predictive value of tenography in combination with arthrography was found to be 100% for the diagnosis of lateral ankle ligament ruptures. The findings from previous studies, along with the findings of the current study, indicate that peroneal tenography is a reliable method to diagnose lateral ankle ligament injuries.

Strengths and limitations

This study capitalised on the usefulness of the peroneal tenography procedure to identify and treat peroneal tendon disorders. Considering the sparse literature available surrounding this procedure, the applicability of peroneal tenography in the diagnosis of peroneal tendon pathology was clearly demonstrated. There was a trend for significant pain relief post procedure, although not statistically significant.

The study was limited by its single-centre design and small sample size. Out of the total sample size of 27, only half the cohort underwent surgery, making it difficult to draw significant conclusions owing to the small sample size. This study did not investigate ankle MRI with suspicion of peroneal tendon injuries which might have improved by physiotherapy. Data for MRI and peroneal tenography were retrieved from the written report instead of a blinded review of images and saved radiographs by standardised musculoskeletal radiologists and foot and ankle surgeons, respectively. Although the outcome was reported in terms of pain relief, the study could have been improved by assessing functionality using a foot and ankle scoring system such as the American Orthopaedic Foot and Ankle Society Score [30].

A recommendation for future research will be that the diagnostic and therapeutic comparison of ultrasound and peroneal tenography has not been explored yet, which could help combine the merits of both techniques for the early management of peroneal tendon disorders. Although there are few case series about the use of platelet-rich plasma in the treatment of peroneal tendinopathy, randomised control trials should be conducted to ascertain its clinical efficacy.

## Conclusions

Peroneal tenography is a safe procedure that can be performed under local anaesthetic in the theatre setting with available inventories, including image intensifiers. This could help reduce the incidence of surgery in patients with chronic ankle pain and could be applied in clinical practice for better patient outcomes. This study found that peroneal tenography was more sensitive and specific than MRI to identify peroneal pathology when compared to intraoperative findings. It also found that peroneal tenography was therapeutically effective for pain management. These findings indicate that peroneal tenography should be highly considered as an intermediary modality prior to surgery when MRI is inconclusive or vague.
